# Water motion and pH jointly impact the availability of dissolved inorganic carbon to macroalgae

**DOI:** 10.1038/s41598-022-26517-z

**Published:** 2022-12-19

**Authors:** Rebecca K. James, Christopher D. Hepburn, Daniel Pritchard, Derek K. Richards, Catriona L. Hurd

**Affiliations:** 1 Department of Botany, University of Otago, Dunedin, New Zealand; 2 Department of Geosciences, Environment & Society Department, Université Libre de Bruxelles, Brussels, Belgium; 3 Department of Marine Science, University of Otago, Dunedin, New Zealand; 4 Coastal People Southern Skies Centre of Research Excellence, Dunedin, New Zealand; 5 Institute for Marine and Antarctic Studies (IMAS), University of Tasmania, Hobart, TAS Australia

**Keywords:** Climate-change ecology, Plant ecology, Marine biology

## Abstract

The supply of dissolved inorganic carbon to seaweeds is a key factor regulating photosynthesis. Thinner diffusive boundary layers at the seaweed surface or greater seawater carbon dioxide (CO_2_) concentrations increase CO_2_ supply to the seaweed surface. This may benefit seaweeds by alleviating carbon limitation either via an increased supply of CO_2_ that is taken up by passive diffusion, or via the down-regulation of active carbon concentrating mechanisms (CCMs) that enable the utilization of the abundant ion bicarbonate (HCO_3_^−^). Laboratory experiments showed that a 5 times increase in water motion increases DIC uptake efficiency in both a non-CCM (*Hymenena palmata,* Rhodophyta) and CCM (*Xiphophora gladiata,* Phaeophyceae) seaweed. In a field survey, brown and green seaweeds with active-CCMs maintained their CCM activity under diverse conditions of water motion. Whereas red seaweeds exhibited flexible photosynthetic rates depending on CO_2_ availability, and species switched from a non-CCM strategy in wave-exposed sites to an active-CCM strategy in sheltered sites where mass transfer of CO_2_ would be reduced. 97–99% of the seaweed assemblages at both wave-sheltered and exposed sites consisted of active-CCM species. Variable sensitivities to external CO_2_ would drive different responses to increasing CO_2_ availability, although dominance of the CCM-strategy suggests this will have minimal impact within shallow seaweed assemblages.

## Introduction

Temperate macroalgal (seaweed) assemblages (e.g. kelp forests) form diverse habitats that provide important ecosystem services. They contribute significantly to primary production, provide a habitat for fish and marine invertebrates, are an important driver of carbon and nutrient cycling, and provide coastal protection services by attenuating waves^1–4^. Dissolved inorganic carbon (DIC) uptake by macroalgae is a fundamental process in photosynthesis, with the uptake mechanism and its rate depending upon the carbon requirements of the individual and the availability of DIC at the algal surface^5,6^. Most of the worlds primary producers are reliant on CO_2_ for photosynthesis, however, CO_2_ currently makes up only 1% of the DIC in seawater (~ 10–20 µM)^7,8^. Approximately 65% of marine macroalgal taxa^9^, therefore, supplement their carbon requirements with the highly abundant HCO_3_^−^ (~ 1700–2100 µM)^7,8^. While CO_2_ passively diffuses across the cellular membranes of macroalgae, a carbon dioxide concentrating mechanism (CCM) is required for the utilisation of HCO_3_^−^^5,10–12^. Several types of CCM exist for macroalgae, including the external dehydration of HCO_3_^−^ to CO_2_ by carbonic anhydrase on the outside of the cell membrane^5^ and anion-exchange proteins that actively pump HCO_3_^−^ into the cell where HCO_3_^−^ is dehydrated by internal carbonic anhydrase^10^. The active conversion of HCO_3_^−^ to CO_2_ via a CCM allows algae to sustain a high concentration of CO_2_ at the site of RUBISCO where it becomes fixed for photosynthesis, independent of the concentration of CO_2_ within the bulk seawater. Although there is an energetic cost to running a CCM, these costs are relatively economical when considering the cost of oxygenase activity by RUBISCO under carbon-limiting conditions^5,6,13^. Macroalgae that do not operate an active CCM and rely solely on the diffusive supply of CO_2_ from the water column are termed ‘non-CCM’.

The availability of DIC for carbon uptake by macroalgae is controlled by the rate of mass transfer of DIC to the macroalga’s surface and, if a CCM is present, the reaction rates of the CCM^14–17^. A key factor affecting the mass transfer rates of solutes to the surface of algae is the concentration of the solute within the bulk seawater. Lowering seawater pH caused by the sustained uptake of anthropogenically produced carbon dioxide (CO_2_), termed ocean acidification^8,18,19^, is causing a rapid change in the seawater carbonate chemistry^8^. By 2100 it is estimated that dissolved CO_2_ concentrations will increase by 200% and bicarbonate (HCO_3_^-^) by 9% in the world’s oceans^8,18^. An increase in the concentration of CO_2_ within seawater would theoretically increase the mass transfer rate of CO_2_ to the algal surface, and thus, may affect photosynthesis of both non-CCM and CCM species^7,20–22^.

Local physical conditions like water motion also impact the mass transfer rates of solutes (i.e. DIC). Mass transfer rates of solutes are greater in fast compared to slow flows because of a faster rate of advection and thinner diffusive boundary layers (DBLs)^15,23^. Also, oscillatory flow and wave action reduce DBL thickness and create turbulence at the macroalgal surface, which can lead to more frequent replenishment of the solutes at the blade compared to wave-sheltered sites or sites dominated by unidirectional flow^15,24,25^. Given the preferential use of CO_2_ by algae^5,26^ and that fluxes of DIC (including CO_2_) to macroalgae would be greater in sites with oscillatory water motion or higher flows, water motion may cause a higher ratio of CO_2_ versus HCO_3_^-^ uptake in wave-exposed versus -sheltered habitats. Quantifying the effect of water motion and changes in the concentration of CO_2_ on carbon uptake in macroalgae is a necessary step before we can understand how global changes in the seawater carbon chemistry (due to ocean acidification) will impact natural macroalgal assemblages.

Theoretically, an increase in the availability of CO_2_ within seawater, either through water motion or an increase in the concentration, should positively affect the photosynthesis of both CCM and non-CCM macroalgae by increasing diffusive supply and enabling the down-regulation of CCMs, or for those species currently limited by carbon, releasing them from DIC limitation^5,27^, i.e. species in slow flow habitats. Evidence for this beneficial effect of increased CO_2_ supply remains limited, however, with variable responses of macroalge being observed in laboratory experiments aimed at studying the effects of ocean acidification and the increase in the concentration of CO_2_^28–32^. These variable responses are most likely linked to differing levels of DIC limitation between individuals^21,33^ and their strategies of carbon-use. Carbon-use strategies of macroalgae relate to the carbon-demands of individuals, which in turn is affected by the physical environment. The effect of light on the carbon physiology of algae can be directly observed in the distribution of carbon uptake strategies within natural macroalgal assemblages: Macroalgae that rely solely on the diffusive uptake of CO_2_ (non-CCM species) are positively correlated with increasing depth, due to the lower carbon requirements of individuals in deeper, low light environments and there being less available energy to operate a CCM^20,34–36^. Whether water motion impacts the distribution of carbon-use strategies in macroalgal assemblages in a similar way to light has not yet been investigated. However, a positive relationship between growth rates and water motion is often observed in macroalgal assemblages^23,37–39^, which would also result in higher carbon-demands. The extent that water motion impacts uptake of DIC by macroalgae and how much it contributes to the distribution of carbon-use strategies in natural macroalgal assemblages remains unknown but would indicate the importance of water motion on carbon-uptake in natural assemblages.

To investigate how greater availability of CO_2_ impacts carbon uptake in individual macroalgae and entire assemblages, we examined carbon-use of macroalgae in both a natural field setting and within controlled laboratory experiments. The natural distribution of carbon-use strategies (CCM, non-CCM and calcareous) in present-day macroalgal assemblages was quantified using carbon stable isotope signatures (δ^13^C)^11^ analysed from macroalgae within replicate wave-sheltered and wave-exposed *Macrocystis pyrifera* (Ochrophyta, Phaeophyceae) kelp forests. To further examine how DIC uptake efficiency and the photosynthetic maximum of macroalgae is affected by both slow and fast water flows and an increase in the concentration of CO_2_ from lowering oceanic pH, a full-factorial DIC uptake laboratory experiment was conducted on a non-CCM red macroalga (*Hymenena palmata,* Rhodophyta) and CCM brown (*Xiphophora gladiata,* Ochrophyta, Phaeophyceae) macroalga. Slow and fast water flows were simulated by low and high mixing, while ambient pH (pH = 8.1) and low pH conditions (pH = 7.6; predicted end of the century pH under the IPCC’s RCP 8.5 emissions scenario^18^) were created to determine the effect of the higher proportion of CO_2_ in DIC under low pH conditions.

We hypothesised that: 1. Non-CCM macroalgae that are constrained to diffusive uptake of CO_2_ will be more abundant in wave-exposed habitats, which have higher mass-transfer rates for CO_2_ due to increased mixing and thinner diffusive boundary layers, compared to wave-sheltered habitats and; 2. Due to the increased availability of CO_2_ under reduced pH and fast flow conditions, both CCM and non-CCM species of macroalgae would more efficiently take up DIC for photosynthesis than when exposed to present-day, slow flow conditions, but a larger response would be seen in the non-CCM species that are dependent solely on CO_2_. Understanding how variations within DIC availability alter the carbon-use of macroalgae and their entire assemblages is fundamental for understanding coastal carbon cycling and productivity, in addition to helping elucidate how macroalgal assemblages will respond to future changes in the oceanic carbon chemistry.

## Results

### Site information

Gypsum blocks were used to compare the rates of mass transfer at the wave-exposed and wave-sheltered sites. The average mass transfer rates were over two-times higher at the wave-exposed field sites compared to at the wave-sheltered sites (Table 1). Light attenuation, temperature, salinity and nitrate concentrations did not significantly differ between the wave-sheltered and -exposed sites, although there was slightly higher concentration of phosphate and ammonium at the wave-exposed sites (Table 1).

**Table 1:** Abiotic site measurements of the three wave-exposed and four wave-sheltered sites.

Site measurements	Wave exposed	Wave sheltered
Mass transfer (g_dwt_ h^−1^)	3.64 ± 0.42	1.76 ± 0.2
Light attenuation	0.3 ± 0.06	0.32 ± 0.03
Temperature (°C)	11.0 ± 0.62	10.1 ± 0.62
Salinity (psu)	35 ± 0.01	35 ± 0.01
Phosphate (µM)	0.31 ± 0.02	0.26 ± 0.01
Ammonium (µM)	1.17 ± 0.14	0.65 ± 0.05
Nitrate (µM)	3.65 ± 0.03	3.6 ± 0.04

Values represent mean ± 95% confidence interval.

### Assemblage structure

**Figure 1:**
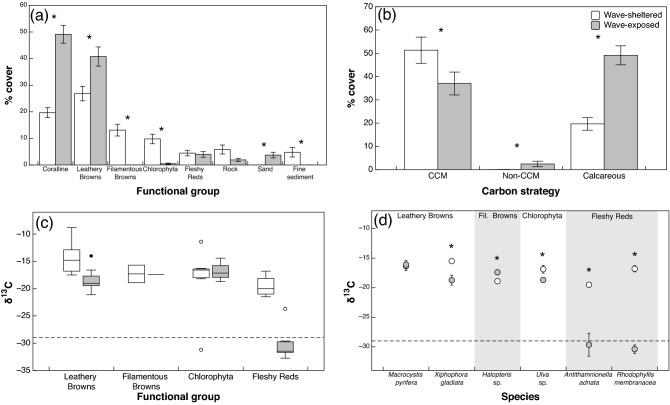
(**a**) Percent cover of functional groups at wave-exposed (grey) and wave-sheltered (white) sites in macroalgal assemblages. Bars represent mean ± 1 SE (n_(exposed)_ = 3, n_(sheltered)_ = 4). (**b**) Percent cover of CCM, non-CCM and coralline species at the wave exposed and wave sheltered assemblages. (**c**) ^13^C signatures (δ^13^C) of species present at the two water motion regimes. Species are grouped into functional groups. δ^13^C values that fall below the dashed line at − 29‰ are considered species that solely use carbon dioxide (CO_2_). (**d**) ^13^C signatures for individual species at the wave-exposed and sheltered sites. Points represent mean ± 1 SE (n = 3 to 21). Asterisks (*) indicate a significant difference between water motion regimes (P < 0.05), tested with a Nested ANOVA (**a–c**), or one-way ANOVA (**d**).

The difference in the assemblage structure between the wave-exposed and -sheltered sites was investigated by conducting community surveys at all sites in summer (February). Coralline algae (crustose and articulated, Rhodophyta) and leathery browns (Ochropyta, Phaeophyceae) were the dominant functional groups observed at the wave-exposed sites covering 49% (SE = 3.4, n = 3) and 40% (SE = 3.6, n = 3) of reef surfaces respectively (Fig. 1a). These groups were also dominant at the wave-sheltered sites with coralline algae covering 20% (SE. = 1.9, n = 4) and leathery browns covering 27% (SE = 2.7, n = 4) of the rock substratum (Fig. 1a). The abundance of the greens (Chlorophyta) and filamentous browns (Ochropyta, Phaeophyceae) was significantly higher at the wave-sheltered sites than at the wave-exposed sites (ANOVA, greens: F_1,5_ = 25.71, P < 0.001; filamentous browns: F_1,5_ = 40.39, P < 0.001). There was no significant difference in the abundance of fleshy red algae (Rhodophyta) with wave-exposure. There was almost double the benthic cover of bare substrate at the wave-sheltered sites, which was composed of 6% (SE. = 1.7, n = 4) rock and 5% (SE. = 1.8, n = 4) fine sediment, compared to the 2% (SE. = 0.6, n = 3) rock and 4% (SE. = 1.1, n = 3) sand coverage at the wave-exposed sites (Fig. 1a).

### Prevalence of carbon-use strategies in sub-canopy assemblages

Carbon stable isotope signatures (δ^13^C) were used to indicate whether macroalgae had an active CCM are were using HCO_3_^−^ (δ^13^C ≥ − 29‰) or were not using a CCM and relied solely on diffusive CO2 uptake (δ^13^C < − 29‰)^11,20,34,40,41^.The abundance of non-CCM species was less than 3.7% (SE = 0.99, n = 3) of the macroalgal cover within wave-exposed assemblages (Fig. 1b), however, this was significantly more than the wave-sheltered sites where there was only one non-CCM species present at one of the sites (*Caulerpa brownii,* Chlorophyta*)*, making up 0.05% of the total macroalgal cover at sheltered sites (ANOVA, F_1,5_ = 59.3, P < 0.001). The majority of macroalgae possessed δ^13^C value that indicated an active CCM at both the wave-sheltered (mean = 56.3%, SE = 2.33, n = 4) and the wave-exposed sites (mean = 41.49%, SE = 2.83, n = 3; Fig. 1b). Calcareous coralline algae and bare substrate made up the remaining benthic cover (Fig. 1a,b).

Three species at the wave-exposed sites, all belonging to the fleshy red functional group, consistently exhibited δ^13^C less than − 29‰ and were thus classified as non-CCM macroalgae: *Ectophora depressa* (formerly *Callophyllis depressa*)*, Hymenena durvillei* and *Streblocladia glomerulata* (Fig. 1c). Both *Rhodophyllis membranacea* (formerly *Rhodophyllis gunnii*) and *Antithamnionella adnata* were fleshy red algae that exhibited varying carbon-use strategies (Fig. 1d). At the wave-exposed sites*, A. adnata* and *R. membranacea* exhibited δ^13^C below − 29‰ indicating sole CO_2_ use, however at wave-sheltered sites the δ^13^C of the same species were significantly more positive, up to − 18.8‰ for *A. adnata* and − 15.8‰ for *R. membranacea* suggesting active uptake of HCO_3_^−^ (Fig. 1d)*.* The green macroalgae *Caulerpa brownii* was the only species with a δ^13^C value more negative than − 29‰ at the wave-sheltered sites, and this species was observed at just one of the four wave-sheltered sites.

Four of the six species that occurred at both the wave-sheltered and wave-exposed sites had significantly lower δ^13^C at the wave-exposed sites (Fig. 1d; ANOVA: *Xiphophora gladiata*: F_1,13_ = 12.17, P = 0.004; *Ulva* sp*.*: F_1,13_ = 24.41, P < 0.001; *A. adnata*: F_1,7_ = 5.57, P = 0.05; *R. membranacea.*: F_1,7_ = 125.78, P < 0.001). The brown *Halopteris* sp. had a significantly higher δ^13^C value at the wave-exposed sites (− 17.4‰) compared with the individuals at the wave-sheltered sites (− 18.9‰; ANOVA: F_1,4_ = 7.60, P = 0.05). There was no significant difference between the δ^13^C values of *Macrocystis pyrifera* at wave-exposed versus wave-sheltered sites (Fig. 1d).

### DIC uptake laboratory experiment

**Figure 2:**
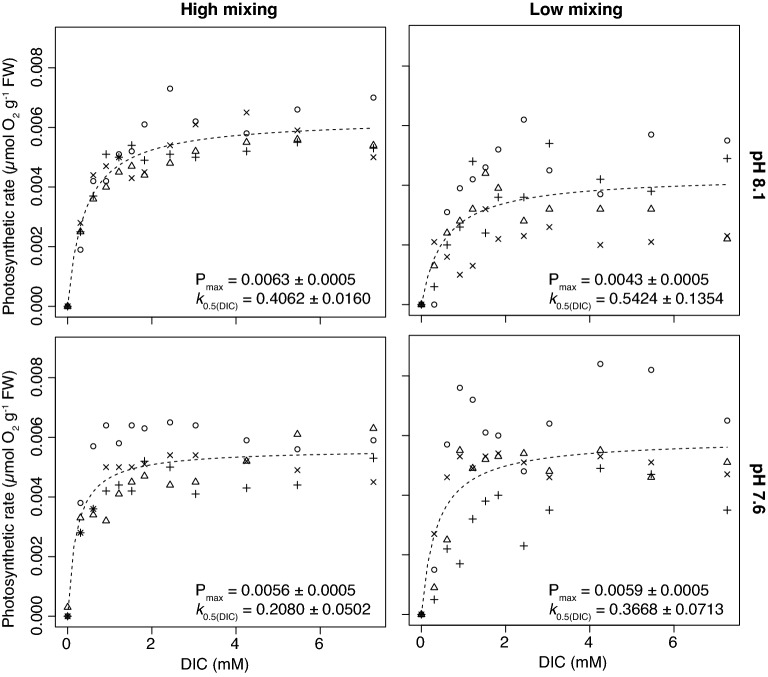
Photosynthetic rates (P) of *Hymenena palmata* (non-CCM) at increasing concentrations of DIC at high (left graphs) and low (right graphs) mixing treatments and at a pH of 8.1 (top graphs) and low pH of 7.6 (bottom graphs). Different shaped points represent the individual replicates (n = 4) with the dashed line indicating the fitted Michaelis–Menten curve ($P=\frac{{P}_{max}\left([DIC]\right)}{{k}_{0.5}+[DIC]})$. Values of P_max_ and *k*_0.5(DIC)_ listed on the figure are means ± SE.

**Figure 3:**
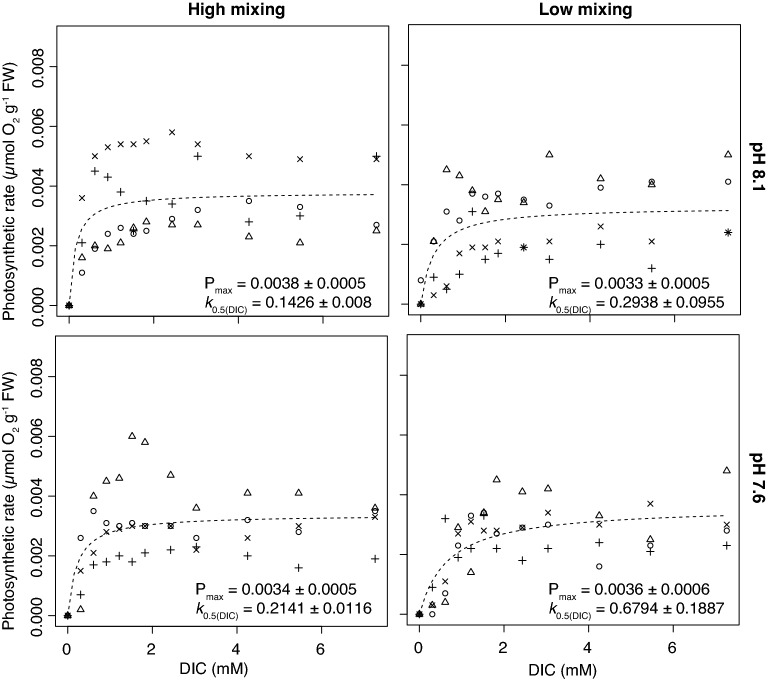
Photosynthetic rates (P) of *Xiphophora gladiata* (CCM) at increasing concentrations of DIC at high (left graphs) and low (right graphs) mixing treatments and at a pH of 8.1 (top graphs) and low pH of 7.6 (bottom graphs). Different shaped points represent the individual replicates (n = 4) with the dashed line indicating the fitted Michaelis–Menten curve ($P=\frac{{P}_{max}\left([DIC]\right)}{{k}_{0.5}+[DIC]}$). Values of P_max_ and *k*_0.5(DIC)_ listed on the figure are means ± SE.

**Figure 4:**
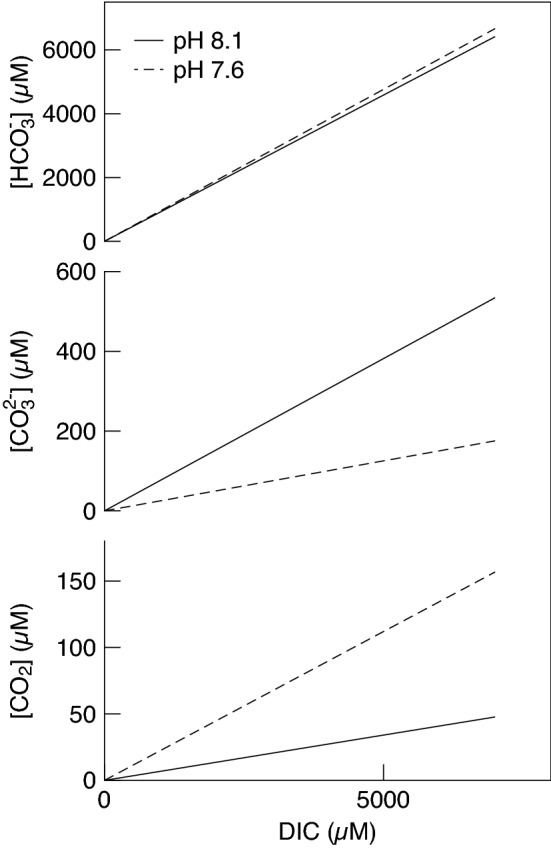
The concentrations of CO_2_, CO_3_^2−^ and HCO_3_^−^ in the ambient pH 8.1 and the low pH 7.6 treatment from the DIC uptake laboratory experiments. The low pH conditions cause a shift in the speciation to a higher concentration of CO_2_ and a lower concentration CO_3_^2−^ within the seawater. Values were calculated using the ‘seacarb’ package^42^ in R, using a temperature of 12 °C and a salinity of 35 psu, with the assumption that the photosynthesis of the ~ 1 g individuals during the incubations did not have a significant impact on the carbonate chemistry within the chambers.

In order to understand how the interactive effects of water motion and an increase in the concentration of CO_2_ caused by lowering pH affect CCM and non CCM species, a photosynthesis vs. DIC uptake experiment was conducted under constant light conditions. Water motion, which was simulated by creating a high- or low-mixed environment, and pH both significantly affected the uptake efficiency of DIC (*k*_0.5(DIC)_) for the non-CCM *H. palmata,* and the CCM species *X. gladiata* (Figs. 2, 3). At the present-day pH of 8.1, the *k*_0.5_ of the non-CCM *H. palamata* was significantly lower in the high-mixed treatment compared with the low-mixed treatment (Simultaneous *t* tests, t = 7.9, P < 0.001), which corresponded to a 25% increase in its DIC uptake efficiency (Table 2). The effect of mixing was even more significant within the high CO_2_, low pH treatment of 7.6 (Simultaneous *t* tests, t = 6.7, P < 0.001), with the DIC uptake efficiency of *H. palmata* being 43% more efficient in high- compared with low-mixed conditions (Table 2). The lower seawater pH treatment had a concentration of CO_2_ that was 230% higher than that of the ambient pH (8.1) treatment (Fig. 4) and had a similar effect as water motion on increasing the DIC uptake efficiency of *H. palmata.* The k_0.5_ of *H. palmata* reduced by 49% from 0.41 mM (SE = 0.016, n = 4; Table 2) in the pH 8.1 treatment to 0.21 mM (SE = 0.05, n = 4; Table 2) at a pH of 7.6 under high mixing. In low-mixed conditions the high CO_2_, low pH still resulted in a significantly lower *k*_0.5_ value (Simultaneous t-tests, t = 7.6 P < 0.001), however, the difference was less extreme with a 32% difference between the pH 8.1 and 7.6 treatments (Table 2). The photosynthetic maximum (P_max_) of *H. palmata* was significantly higher in the high mixed versus the low-mixed treatment at pH 8.1 (Simultaneous *t* tests, t = 2.88, P = 0.03) but there was no significant difference in the P_max_ at the lower pH treatment of 7.6 even though the CO_2_ concentration was 230% higher (Table 2).

**Table 2:** Carbon uptake efficiency (k_0.5_) and photosynthetic maximum (P_max_) of a non-CCM red (*Hymenena palmata*) and CCM brown species (*Xiphophora gladiata*) under high- and low-mixing conditions and high (8.1) and low (7.6) pH.

Species	Mixing	pH	*k*_0.5_ (mM_DIC_)	P_max_ (μmol O g^−1^ FW s^−1^)
*Hymenena palmate* (Non-CCM)	High	8.1	0.4062 ± 0.0160^a^	0.0063 ± 0.0005^a^
	Low	8.1	0.5424 ± 0.1354^b^	0.0043 ± 0.0005^b^
	High	7.6	0.2080 ± 0.0502^c^	0.0056 ± 0.0005^a,b^
	Low	7.6	0.3668 ± 0.0713^a^	0.0059 ± 0.0005^a,b^
*Xiphophora gladiata* (CCM)	High	8.1	0.1426 ± 0.008^i^	0.0038 ± 0.0005^i^
	Low	8.1	0.2938 ± 0.0955^j^	0.0033 ± 0.0005^i^
	High	7.6	0.2141 ± 0.0116^k^	0.0034 ± 0.0005^i^
	Low	7.6	0.6794 ± 0.1887^l^	0.0036 ± 0.0005^i^

Values represent mean ± SE, n = 4.

Simultaneous *t* tests were used to test for significant differences between the water motion and pH treatments within each species, with a p-value < 0.05 indicating a significant difference.

Different lowercase letters indicate significant differences between values, while the same letter indicates there is no significant difference.

The DIC uptake efficiency of *X. gladiata* also significantly increased in the high mixing treatment (Simultaneous *t* tests, pH 8.1: t = 18.4, P < 0.001; pH 7.6: t = 40.1, P < 0.001), with a 52 and 69% reduction in the k_0.5_ value in the pH 8.1 and 7.6 treatments respectively (Table 2). The response of the DIC uptake efficiency of *X. gladiata* to low pH, high CO_2_ conditions contrasted, however, to that of *H. palmata*, with the uptake efficiency of *X. gladiata* being negatively impacted within the low pH, high CO_2_ treatment*.* Within the high-mixed treatment, the *k*_0.5_ of *X. gladiata* increased from 0.14 mM to 0.21 mM (Table 2), which corresponded to a significant 50% decrease in uptake efficiency (Simultaneous *t* tests, t = 8.75, P < 0.001). Within the low-mixed treatment, the negative effect of the low pH treatment was exacerbated even more, showing a 131% decrease in DIC uptake efficiency of *X. gladiata* (Simultaneous t-tests, t = 33.27, P < 0.001; Table 2). Although the DIC uptake efficiency of *X. gladiata* was significantly affected by both mixing and pH (Table 2), there was no significant effect to its P_max_ (Fig. 3; Table 2)*.*

The photosynthesis of both algal species saturated well-below the concentration of DIC in natural seawater of ~ 2 mM: that of *X. gladiata* saturated at a DIC concentration between 0.3 and 1.36 mM (Fig. 3) and the photosynthesis of *H. palmata* saturated between 0.41 and 1.09 mM DIC (Fig. 2). A higher variability was observed in the low-mixed compared to the high-mixed treatment for both species. This variability is likely to be a consequence of unstable readings from the O_2_ sensor in the low-mixed environment.

## Discussion

Water motion regulates the uptake of DIC by macroalgae, with increased mixing enabling more efficient uptake of DIC for photosynthesis. This physiological response was evident in laboratory experiments under controlled conditions, however, the effect on natural macroalgal assemblages in situ was less clear. It was hypothesized that a greater availability of CO_2_ at wave-exposed sites would lead to more non-CCM macroalgae that rely solely on diffusive CO_2_ uptake compared to wave-sheltered sites. A greater abundance and diversity of non-CCM macroalgae was found at the wave-exposed versus the wave-sheltered sites, however, this accounted for only 3 species with a total benthic cover of 3.7%. When we examine this in situ response at the functional group level, however, we observe that fleshy red macroalgae, which are a group where the non-CCM uptake strategy is most common^11^, respond strongly to the water motion regime compared to brown and green macroalgae. The varying responses between functional groups in the laboratory compared to the field suggest that water motion is but one of many factors regulating the uptake of DIC by macroalgae in situ. Alongside water motion, the light regime, nutrient availability, the life strategy of a species and the plasticity of a species carbon-uptake strategy all can influence carbon uptake in natural macroalgal assemblages, making it difficult to predict how entire macroalgal assemblages will respond to changes in the seawater carbon chemistry.

The majority of macroalgal species within the kelp forest assemblages of southern New Zealand possessed values of δ^13^C indicative of an active CCM^11,41^ at both wave-sheltered and -exposed sites. This dominance suggests that the carbon demands of most of the species in these assemblages cannot be sustained by CO_2_ alone and additional DIC from the large HCO_3_^−^ pool is required^5,43^ i.e. the photosynthetic rates of the macroalgae exceed what can be sustained with only diffusive uptake of CO_2_. Active CCMs are almost universal in both brown and green macroalgae, with reports of only a handful of green macroalgal species exhibiting δ^13^C less than − 29 indicating sole-CO_2_ use^11,34,36,44,45^. These non-CCM brown and green macroalgae are typically found in low light environments, either in deep habitats or beneath dense canopies, where the carbon-demands of individuals are lower^20,34,36^. Given browns followed by greens were the dominant functional groups at both the shallow wave-exposed and -sheltered sites, the high abundance of the CCM strategy is expected.

An interesting observation was the difference in within-species variability of the δ^13^C. The dominant canopy forming brown species, *M. pyrifera* showed very little variability in carbon-use between individuals at different sites, a pattern consistent with previous work^24^. *M. pyrifera* has an efficient mechanism of bicarbonate uptake, via an anion-exchange protein ^10,46^, which allows it to maintain high growth rates^24^ irrespective of the availability of CO_2_. Other brown and green species that occurred at both water motion regimes showed more variability in their δ^13^C while still exhibiting HCO_3_^−^ use. Given the sites are constantly subtidal and are all linked by the same body of water with no nearby freshwater inputs, we assume that the ^13^C/^12^C source is the same at all sites^11^. The lower δ^13^C values could suggest downregulation of the CCM, however, ^13^C signatures on their own cannot be used to disentangle the variability in ^13^C signatures that are more positive than − 29‰^11^. Local differences in the light environment within the kelp forest canopy, CO_2_ availability through water motion and differences in the health and size of individuals and their acclimation to the light environment could all contribute to different carbon requirements and variability of the δ^13^C values from the active-CCM species.

The greatest within-species variability of carbon-use was observed in the fleshy red species. The Rhodophyta phylum has the highest abundance of species with δ^13^C less than − 29, with 35.6% of species that have been analysed showing δ^13^C indicative of sole-CO_2_ use^11^. Fleshy red species, *Antithamnionella adnata* and *Rhodophyllis membranacea*, were observed in this study to have highly variable carbon-use strategies, both possessing ^13^C signatures indicative of HCO_3_^−^ use at the wave-sheltered sites, however, having more negative ^13^C signatures at the wave-exposed sites that suggested the sole use of CO_2_. Modulation of CCM activity has been extensively studied within the green unicellular alga *Chlamydomonas reinhardtii*^47–49^ and has also been identified in *Ulva lactuca*^50^. Although modulation of the CCM by algae from the Rhodophyta phylum has not yet been examined, the species we observed seem to possess an ability to optimise their carbon-use depending upon the external CO_2_ availability. Such a mechanism would allow these algae to minimise their energy expenditure, but also makes them sensitive to the external CO_2_ availability.

By increasing rates of advective transport and reducing the thickness of boundary layers^25,51^, we observe in the laboratory experiments that water motion allows macroalgae to take up DIC at a faster rate. This physiological effect of an increase in the availability of DIC does not appear to strongly impact brown and green macroalgae with active CCMs, as they are likely to be saturated for carbon at current DIC concentrations and instead their photosynthetic rate is more limited by other factors, such as light or nutrients. We observed this DIC saturation in the experiments with *X. gladiata,* which reached its maximum photosynthetic rate (P_max_) at DIC levels below half that of present-day DIC concentrations even at saturating light levels. The P_max_ of the CCM species *X. gladiata* remained constant across water motion and pH treatment conditions, even when those conditions would theoretically reduce the availability of DIC at the alga’s surface. This suggests that *X. gladiata* does not significantly down-regulate the activity of its CCM when the availability of DIC decreases, instead maintaining a high photosynthetic rate under a wide range of conditions. In contrast, the non-CCM species, *Hymenena palmata* did show evidence of carbon limitation in terms of its P_max_. Under present-day pH conditions (pH 8.1), *H. palmata* (non-CCM) exhibited a lower P_max_ within the low-mixed compared to the high-mixed treatment, a finding consistent with Kübler and Dudgeon^9^ who showed with a model that thick boundary layers can limit net photosynthetic rates of non-CCM macroalgae. Interestingly, when the proportion of CO_2_ in the total DIC was increased in the low-pH treatment, the P_max_ of *H. palmata* within the low-mixed treatment matched that of the high-mixed treatment. Unlike *X. gladiata,* it appears that the photosynthetic rate of this non-CCM red species depends upon the availability of CO_2_, indicating a greater sensitivity to external CO_2_ availability than the brown macroalgae with an active CCM.

It was hypothesised that regulation of CO_2_ uptake by water motion would be counteracted by a lower pH that increases the concentration of CO_2_. This positive response to increasing CO_2_ availability, however, is dependent on the species sensitivity to the external CO_2_ concentration. This sensitivity seems to be related to the absence of a CCM and the local environment that the organism inhabits (i.e., habitats with low mixing). The low pH treatment did, however, have an alternative negative effect on the CCM species, *X. gladiata*, with this species exhibiting a decrease in DIC uptake efficiency in the lower pH treatment compared to the ambient pH, both in high- and low-mixed conditions. Johnston and Raven^52^ observed a similar finding when *Fucus serratus* (brown) showed a reduced affinity of CO_2_ when grown in high CO_2_ conditions^52^. This could be attributed to the CCM or another physiological process of the algae being repressed, however, it is unclear whether this is caused by the low pH or the high CO_2_^32,52^. Indeed, a low pH means that there is an increase in the [H^+^] within the seawater. As H^+^ plays a vital role in the regulation of cellular homeostasis, an increase in [H^+^] could impact metabolic processes and CCM activity of certain species of macroalgae^32^. However, not all CCM macroalgal species show this response and other species with CCMs have shown significantly positive^32^ or no photosynthetic or growth response^46^ to increasing *p*CO_2_ and lowering pH.

Water motion influences several factors in the coastal environment, impacting not only solute exchange but also the sediment dynamics, the light environment, temperature and grazing^14,25^. Coralline algae were significantly more abundant at the wave-exposed sites compared with the wave-sheltered sites. This is most likely due to more fine sediment and turfing algae being present at the sheltered sites, where they were observed smothering the low-lying encrusting coralline algae. Coralline algae typically possess CCMs and will, to varying degrees, also assimilate CO_2_ via diffusion^40^, so are not expected to be limited by DIC. Bergstrom et al.^40^ investigated the carbon uptake response of crustose coralline algae to OA and found that only one out of six coralline algae species down-regulated its CCM under elevated *p*CO_2_, suggesting that most coralline species will not have an obvious positive benefit from increasing CO_2_ availability.

Given the contrasting responses to changing CO_2_ availability in both the field observations and the laboratory experiments, we provide evidence that macroalgal species have differing levels of sensitivity to the external CO_2_ availability. In general, browns and green macroalgae with CCMs are resilient to changing CO_2_ availability and maintain an active CCM in a wide range of conditions, while red fleshy algae that show non-CCM behaviour are more sensitive. This sensitivity indicates that within present-day low-mixed environments, large diffusive boundary layers could impede the delivery of CO_2_ to the surface of non-CCM algae. We suggest that the distribution of non-CCM species in shallow coastal areas is in part regulated by water motion. However, this probably only has a minor effect on the overall structure of shallow macroalgal assemblages in present day CO_2_ conditions in southern New Zealand, as the majority of algae in both the wave-exposed and wave-sheltered sites possess values of δ^13^C indicative of an active CCM^20^. In deeper, low-light field sites that are dominated (> 80%) by non-CCM species (e.g. southeastern Tasmania^34^), however, water motion is likely to strongly regulate the distribution of macroalgal species. Macroalgal species that currently experience carbon limitation, and which are sensitive to external CO_2_ availability for carbon acquisition, could benefit from predicted increases in seawater concentration of CO_2_ caused by the increase of atmospheric CO_2_ from anthropogenic emissions^53^. Although since the majority of macroalgal species possess a CCM^11,41^ and do not appear to be limited under the present-day availability of DIC, a significant change in community structure is unlikely.

## Methods

### Site information

**Figure 5:**
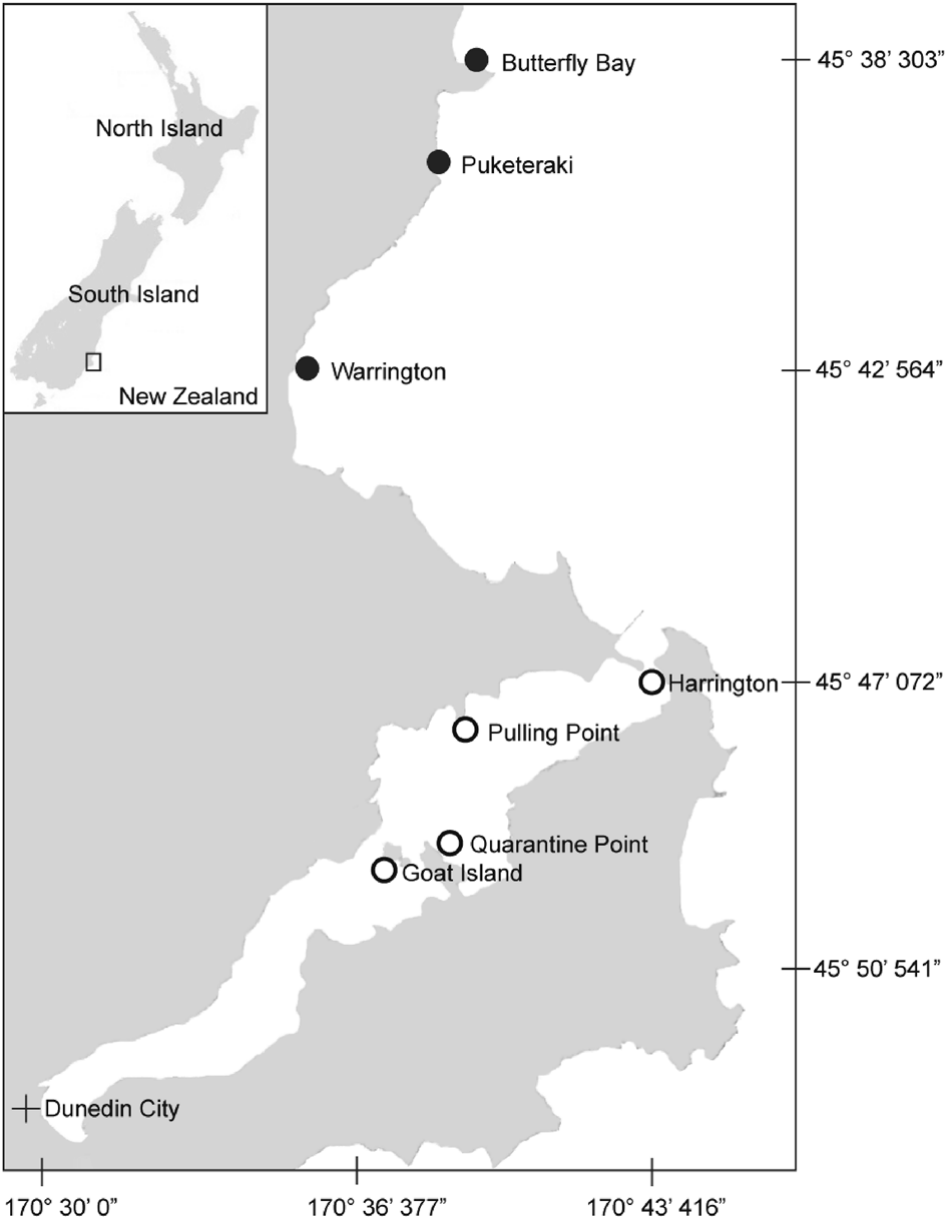
Location of study sites along the East Otago coast and within Otago Harbour, New Zealand. Wave-exposed sites are indicated by black circles and wave-sheltered sites are indicated by white circles with a black outline. Map created using R version 4.2.1 with the ‘rdgal’ package^56^, and using the NZ Coastlines shapefile from the Topo50 map series of Land Information New Zealand (LINZ)^57^.

Surveys of the macroalgal assemblages and tissue collections were conducted within kelp forest habitats between 3 and 5 m below mean low water on the south-eastern coast of the South Island of New Zealand (45°45′27"S, 170°41′37"E). Four wave-sheltered sites within Otago Harbour and three wave-exposed sites along the open coast just north of the harbour were selected (Fig. 5**).** All sites are linked by the same bulk water, and there are no significant freshwater outflows within the area that might influence one site differently. *Macrocystis pyrifera* (Ochropyta, Phaeophyceae, Laminariales) was the dominant kelp canopy at all sites. The presence of the bull kelp *Durvillaea* spp. (Ochrophyta, Phaeophyceae, Fucales) was used as a biological indicator of wave-exposure at the sites, as this species is exclusive to wave-exposed shores^24^.

The rate of dissolution of gypsum blocks was employed to obtain a proxy for the rate of mass transfer of solutes at the study sites^54,55^. Dissolution of gypsum blocks is mediated by the thickness of boundary layers and is driven by advection processes, thereby providing a useful assessment of relative differences in mass transfer rates of solutes between the different sites. The gypsum blocks were made following Gerard and Mann^55^ and were deployed by attaching the blocks to an anchored float, where they were positioned 0.5–1 m below the water surface and on the outer edge of the reef at each site. The blocks were deployed on three separate occasions during April 2010 (autumn) for 48–54 h. The hourly dissolution rate was calculated from the before and after dry weight. It was ensured that more than 30% of the original weight of the gypsum blocks remained, so that the rate of dissolution could be considered linear.

Light attenuation, temperature, salinity and nutrient concentrations were measured on three separate days at each of the sites between April and May 2010. To obtain an estimate of the water turbidity at the sites, light attenuation was measured with a LI-COR LI-193 Spherical Quantum Sensor. Single light measurements were taken every meter and the exponential rate of decay of light with depth was calculated as a slope of the linear regression of the natural logarithm on depth^58^. The temperature and light measurements were compared with the extensive light dataset of the area conducted by Pritchard et al. (2013) to ensure the values were representative of the area. Seawater salinity was determined with a Sper Scientific^©^ Salinity Refractometer. Triplicate water samples were collected from the surface at each of the sites in April 2010 and were filtered through Whatman™ GF/C filters. The samples were frozen before the concentrations of nitrate, ammonium and phosphate were analysed on a Lachat QuickChem^©^ 8000 automated ion analyser using standard methods^59^ within a month of collection.

### ***Surveys of macroalgal assemblages and δ***^***13***^***C analysis***

Benthic surveys were carried out at each of the seven sites during February (summer) 2010 and were completed within one month. 20 A4-sized (210 × 297 mm) underwater photo-quadrats were taken at each of the sites, using a Canon Powershot G10 digital camera in an underwater casing. The photo-quadrats were taken from random positions along a 30 m transect that was haphazardly placed parallel to the shore at a depth of 1–3 m. Percent cover of species and functional groups was calculated with the random point count method, with 50 random points overlaid on each of the photo-quadrats using Coral Point Count^60^. Macroalgae were grouped into their phylum or class i.e. Chlorophyta (greens), Rhodophyta (reds), or Ochrophyta, Phaeophyceae (browns). The browns were further split into filamentous or leathery, and the reds split into fleshy and corallines, to take into account the different ecological roles these groups play within a community.

Corresponding tissue samples were taken from the same location on three replicate individuals of each of the macroalgal species observed at the seven sites were collected at the same time as the community surveys. The thallus samples were cleaned of epibionts, rinsed with filtered seawater, and were identified to species level based on their morphology. The tissue was dried to a constant weight at 80 °C and then ground to a fine powder. δ^13^C (‰) was determined with the ground samples using a CE NA1500 Elemental Analyzer (Carlo-Erba instruments) interfaced to a Europa Scientific 20-20 update continuous flow mass spectrometer (Department of Chemistry, University of Otago, New Zealand). Corrections for drift were made automatically every 5 samples from a standard ethylenediamine tetraacetic (EDTA) with a known isotopic ratio. The δ^13^C values were expressed as ‰ and represent the relative ^13^C:^12^C of the sample against a standard (^13^C:^12^C of Pee Dee Belemnite) multiplied by 1000. The macroalgal species present in each of the assemblages surveyed were grouped into one of three carbon-use strategy groups as used by Hepburn et al.^20^: CCM (δ^13^C ≥ − 29‰), non-CCM (δ^13^C < − 29‰) and coralline algae. There are a number of other potential reasons for low δ^13^C in marine macroalgae, including refixation of respired CO_2_, a 10.72‰ more negative δ^13^C of CO_2_ produced from HCO_3_^−^ during intracellular conversion by carbonic anhydrase and exposure to a more negative δ^13^C of external inorganic carbon due to estuarine input^11^. As discussed in Raven et al.^7,11^, these first two cases are unlikely in Rhodophyta macroalgae (the most common non-CCM group in Southern New Zealand), while differences in the δ^13^C of external inorganic carbon between the study sites is improbable due to the sites all being linked by the same bulk water with no estuarine or freshwater outputs in the vicinity.

### DIC uptake efficiency and O_2_ evolution

In a subsequent laboratory experiment, we examined the effect of water motion and pH on DIC uptake by two species, a CCM and non-CCM, that were dominant in the field surveys. Approximately 1 g thalli samples of *Xiphophora gladiata* subsp. *novae-zelandiae* E.L.Rice (brown CCM species) and *Hymenena palmata* (Harvey) Kylin (red, non-CCM species) were collected from the same location on thalli of different replicate individuals at 1–2 m depth from Butterfly Bay, Karitane, Otago (Fig. 5) between April and May 2011 (Autumn). All thalli samples were taken from the top of the sub-canopy, with only a *M. pyrifera* canopy above them. Thalli were kept in seawater in a cool box for transport back to the laboratory 30 min away. At the laboratory, the thalli were gently cleaned of epiphytes and sediment, and were acclimated overnight in filtered seawater to facilitate wound healing. The thalli were stored for up to 4 days in filtered seawater at a temperature of 12 °C with constant bubbling provided by an air bubbler stone. TrueLumen™ 460 nm Actinic T5HO lamps on a 12:12 light dark cycle provided 100 µmol photons m^−2^ s^−1^ of light to the stored samples. The seawater used in the experiments was collected from the Portobello Marine Laboratory (University of Otago) and filtered through a 0.5 μm pore size Filter Pure^®^ polypropylene spun melt filter and then sterilised with an Aquastep^®^ 25 W Ultraviolet Sterilizer.

A full factorial design was used to measure the effect of two levels of pH (8.1 and 7.6) and two levels of water motion (high- and low-mixed conditions) on the uptake of DIC for photosynthesis in *X. gladiata* and *H. palmata,* with 4 replicates. The IPCC’s RCP 8.5 emissions scenario^18,19^ with a pH of 7.6 by 2100 was used as an extreme future ocean acidification scenario. Experiments were conducted in 150 mL chambers under a photosynthetically saturating irradiance of 300 µmol photons m^−2^ s^−1^ at 12 °C. Light was delivered by a Kodak Carousel^®^ S-AV2000 slide projector. To maintain the treatment pH throughout the experiments and thus a constant ratio of CO_2_:HCO_3_, Tris buffer was added to UV filtered seawater at a concentration of 26 mM. Tris buffer has been reported to affect the photosynthesis of the macroalgae *Saccharina latissima* (formerly *Laminaria saccharina*) (Ochrophyta, Phaeophyceae, Laminariales)*,* by interfering with the method of HCO_3_^−^ utilisation this alga possesses^61^. However, the effect of Tris buffer on the photosynthetic ability of *X. gladiata* and *H. palmata* was tested by measuring the O_2_ evolution of individuals (n = 3) in filtered seawater with and without TRIS, and no detectable effect was found.

DIC was removed from the Tris-buffered seawater by lowering the pH to below 3 with HCl, bubbling with nitrogen gas for 4 h and then raising the pH to either 8.1 or 7.6 with fresh NaOH solution. A stirrer bar was used to modify the level of mixing in the O_2_ evolution chambers, which was turned to either a high or low speed to apply a high mixed treatment representing a fast flow environment where boundary layers would be thin or a low-mixed treatment with minimal mixing and where thicker boundary layers would exist. The level of mixing in the chambers was measured by injecting dye into the chambers and measuring the time for the dye to completely disperse into the seawater. The dye was 5 times faster to disperse in the high mixed treatment, indicating a fivefold increase in the mass transfer rate in the high-mixed relative to the low-mixed treatment.

Sixteen individuals of each species were randomly assigned to each of the four treatment combinations, to give four replicates of each treatment. O_2_ concentration of the water was continuously recorded with a fibre-optic Ocean Optics^®^ FOXY–R sensor probe connected to a USB-2000 spectrophotometer. The O_2_ concentration of the DIC free Tris-buffered seawater was lowered to 20–40% before each experimental-run by briefly sparging with nitrogen gas (~ 5 min), to reduce any possible effect of photorespiration by the algae. Oxygen evolution measurements were begun once the level of O_2_ in the medium was stable (i.e. no photosynthesis was occurring), indicating that individuals had used up any internal inorganic carbon stores or remaining DIC in the seawater medium; this took around 10 min. Every 10 min, 0.05 mL of 1 M NaHCO_3_^−^ was injected into the chamber until the total DIC concentration in the seawater medium was 1.825 mM, thereafter 0.1–0.3 mL additions were made until the total calculated concentration of DIC was 7 mM. This resulted in 12 injections with a total incubation time of 120 min. The photosynthetic rate was measured during each DIC addition, which was used to calculate the DIC-saturated maximum photosynthetic rate (P_max_) and DIC uptake efficiency (*k*_0.5,_ the concentration of DIC where photosynthesis is half of P_max_), which is further detailed in the statistical methods section. The alkalinity and concentrations of CO_2_, HCO_3_^−^ and CO_3_^2−^ of the treatment seawater medium at each addition of DIC were calculated using the known parameters of total DIC, temperature (12 °C), salinity (35 psu) and pH (7.6 or 8.1). The calculations were done using the constants of Mehrbach et al. (1973) refitted by Dickson and Millero (1987), with the R package ‘seacarb’^42^ and are presented in Fig. 4.

The oxygen evolution measurements were standardised to the algal fresh weight. A linear regression was applied to these standardised oxygen evolution measurements between each addition of NaHCO_3_^−^ to calculate the photosynthetic rate (µmol O_2_ g^−1^ FW s^−1^) of the individuals at each DIC concentration.

### Statistical methods

Differences in functional group percent cover and the abundance of each carbon-use strategy, were tested between each site and the two levels of water motion with a nested ANOVA using the R statistical software platform^62^. Each site was nested within either the wave-sheltered or wave-exposed water motion classification factors. Sites were considered replicates of the two levels of water motion, giving 4 wave-sheltered replicates and 3 wave-exposed replicates. The variability between the sites was of interest and so a nested ANOVA test was used to provide a test of comparison between the two water motion classifications, but also among the seven sites taking into account the water motion at the sites. One-way ANOVAs were run for the δ^13^C values of any species occurring at both the wave-exposed and wave-sheltered sites, to examine whether they were significantly different between water motion regimes. A p-value equal to or less than 0.05 was considered significant. No transformations of the data to meet the requirements of these parametric tests were required.

A Michaelis–Menten curve was fitted to plots of photosynthetic rate vs DIC concentration. This model is fitted to data by optimising the photosynthetic maximum (P_max,_ the DIC-saturated maximum photosynthetic rate) and DIC uptake efficiency (*k*_0.5,_ the concentration of DIC where photosynthesis is half of P_max_). A maximum likelihood, non-linear mixed effects modelling approach, using the R statistical software platform^62^ and the lme4 package^63^ was used. This approach allowed a single model for each species, whilst accounting for variability in P_max_ and K_0.5_, by including incubation as a random factor. Within species contrasts on P_max_ and *k*_0.5_ of *X. gladiata* and *H. palmata* were made between the four treatment combinations (low or high mixed treatment with ambient or low pH) using simultaneous t-tests, using the R package multcomp^64^.

## Data Availability

Data is available at the 4TU.ResearchData Data repository: https://doi.org/10.4121/16973866.v1.

## References

[1] Duggins DO, Simenstad CA, Estes JA (1989). Magnification of secondary producition by kelp detritus in coastal marine ecosystems. Science.

[2] Hill R (2015). Can macroalgae contribute to blue carbon? An Australian perspective. Limnol. Oceanogr..

[3] Mann KH (1973). Seaweeds: Their productivity and strategy for growth. Science.

[4] Steneck RS (2002). Kelp forest ecosystems: Biodiversity, stability, resilience and future. Environ. Conserv..

[5] Giordano M, Beardall J, Raven JA (2005). CO2 concentrating mechanisms in algae: Mechanisms, environmental modulation, and evolution. Annu. Rev. Plant Biol..

[6] Raven JA, Beardall J (2016). The ins and outs of CO2. J. Exp. Bot..

[7] Raven JA (2002). Seaweeds in cold seas: Evolution and carbon acquisition. Ann. Bot..

[8] Raven J (2005). Ocean Acidification due to Increasing Atmospheric Carbon Dioxide.

[9] Kübler JE, Dudgeon SR (2015). Predicting effects of ocean acidification and warming on algae lacking carbon concentrating mechanisms. PLoS ONE.

[10] Fernández PA, Hurd CL, Roleda MY (2014). Bicarbonate uptake via an anion excange protein is the main mechanism of inorganic carbon acquisition by the giant kelp Macrocystis pyrifera (Laminariales, Phaeophyceae) under variable pH1. J. Phycol..

[11] Raven JA (2002). Mechanistic interpretation of carbon isotope discrimination by marine macroalgae and seagrasses. Funct. Plant Biol..

[12] Raven JA, Cockell CS, De La Rocha CL (2008). The evolution of inorganic carbon concentrating mechanisms in photosynthesis. Philos. Trans. R. Soc. B.

[13] Bidwell RGSS, McLachlan J (1985). Carbon nutrition of seaweeds: Photosynthesis, photorespiration and respiration. J. Exp. Mar. Biol. Ecol..

[14] Hurd CL (2000). Water motion, marine macroalgal physiology and production. J. Phycol..

[15] Hurd CL, Stevens CL, Laval BE, Lawrence GA, Harrison PJ (1997). Visualization of seawater flow around morphologically distinct forms of the giant kelp Macrocystis integrifolia from wave-sheltered and exposed sites. Limnol. Oceanogr..

[16] Smith FAA, Walker NAA (1980). Photosynthesis by aquatic plants: Effects of unstirred layers in relation to assimilation of CO_2_ and HCO_3_- to carbon isotope discrimination. N. Phytol..

[17] Wheeler WN (1980). Effect of boundary layer transport on the fixation of carbon by the giant kelp Macrocystis pyrifera. Mar. Biol..

[18] Hurd CL, Lenton A, Tilbrook B, Boyd PW (2018). Current understanding and challenges for oceans in a higher-CO_2_ world. Nat. Clim. Chang..

[19] Stocker, T. F. *et al.* Technical Summary. *Climate Change 2013: The Physical Science Basis. Contribution of Working Group I to the Fifth Assessment Report of the Intergovernmental Panel on Climate Change* 33–115 (2013).

[20] Hepburn CD (2011). Diversity of carbon use strategies in a kelp forest community: Implications for a high CO_2_ ocean. Glob. Chang. Biol..

[21] Beer S, Koch E (1996). Photosynthesis of marine macroalgae and seagrasses in globally changing CO_2_ environments. Mar. Ecol. Prog. Ser..

[22] Ihnken S, Roberts S, Beardall J (2011). Differential responses of growth and photosynthesis in the marine diatom Chaetoceros muelleri to CO_2_ and light availability. Phycologia.

[23] Gerard VA (1982). In situ water motion and nutrient uptake by the giant kelp Macrocystis pyrifera. Mar. Biol..

[24] Hepburn CD, Holborow JD, Wing SR, Frew RD, Hurd CL (2007). Exposure to waves enhances the growth rate and nitrogen status of the giant kelp Macrocystis pyrifera. Mar. Ecol. Prog. Ser..

[25] Hurd CL (2017). Shaken and stirred: The fundamental role of water motion in resource acquisition and seaweed productivity. Persp. Phycol..

[26] Sültemeyer DF, Miller AG, Espie GS, Fock HP, Canvin DT (1989). Active CO_2_ transport by the green alga Chlamydomonas reinhardtii. Plant Physiol..

[27] Koch M, Bowes G, Ross C, Zhang XH (2013). Climate change and ocean acidification effects on seagrasses and marine macroalgae. Glob. Chang. Biol..

[28] Britton D, Cornwall CE, Revill AT, Hurd CLCL, Johnson CR (2016). Ocean acidification reverses the positive effects of seawater pH fluctuations on growth and photosynthesis of the habitat-forming kelp Ecklonia radiata. Sci. Rep..

[29] Cornwall CE (2012). Carbon-use strategies in macroalgae: Differential responses to lowered ph and implications for ocean acidification. J. Phycol..

[30] Kram SL (2016). Variable responses of temperate calcified and fleshy macroalgae to elevated pCO_2_ and warming. ICES J. Mar. Sci..

[31] Kübler JE, Johnston AM, Raven JA (1999). The effects of reduced and elevated CO_2_ and O_2_ on the seaweed Lomentaria articulata. Plant Cell Environ..

[32] van der Loos LM (2019). Responses of macroalgae to CO_2_ enrichment cannot be inferred solely from their inorganic carbon uptake strategy. Ecol. Evol..

[33] Cornwall CE, Hurd CL (2019). Variability in the benefits of ocean acidification to photosynthetic rates of macroalgae without CO_2_-concentrating mechanisms. Mar. Freshw. Res..

[34] Cornwall CE, Revill AT, Hurd CL (2015). High prevalence of diffusive uptake of CO_2_ by macroalgae in a temperate subtidal ecosystem. Photosynth. Res..

[35] Lovelock CE, Reef R, Raven JA, Pandolfi JM (2020). Regional variation in δ13C of coral reef macroalgae. Limnol. Oceanogr..

[36] Fischer G, Wiencke C (1992). Stable carbon isotope composition, depth distribution and fate of macroalgae from the Antarctic Peninsula region. Polar. Biol..

[37] Stephens TA, Hepburn CD (2014). Mass-transfer gradients across kelp beds influence Macrocystis pyrifera growth over small spatial scales. Mar. Ecol. Prog. Ser..

[38] Kregting LT, Hepburn CD, Savidge G (2015). Seasonal differences in the effects of oscillatory and uni-directional flow on the growth and nitrate-uptake rates of juvenile Laminaria digitata (Phaeophyceae). J. Phycol..

[39] Parker HS (1981). Influence of relative water motion on the growth, ammonium uptake and carbon and nitrogen composition of Ulva lactuca (Chlorophyta). Mar. Biol..

[40] Bergstrom E (2020). Inorganic carbon uptake strategies in coralline algae: Plasticity across evolutionary lineages under ocean acidification and warming. Mar. Environ. Res..

[41] Maberly SC, Raven JA, Johnston AM (1992). Discrimination between C-12 and C-13 by marine plants. Oecologia.

[42] Gattuso, J. P. *et al.* Package ‘Seacarb ’. Preprint at http://cran.r-project.org/package=seacarb (2015).

[43] Raven JA, Beardall J, Giordano M (2014). Energy costs of carbon dioxide concentrating mechanisms in aquatic organisms. Photosynth. Res..

[44] Raven JA, Walker DI, Johnston AM, Handley LL, Kübler JE (1995). Implications of 13C natural abundance measurements for photosynthetic performance by marine macrophytes in their natural environment. Mar. Ecol. Prog. Ser..

[45] Raven JA (1997). Inorganic carbon acquisition by marine autotrophs. Adv. Bot. Res..

[46] Fernández PA, Roleda MY, Hurd CL (2015). Effects of ocean acidification on the photosynthetic performance, carbonic anhydrase activity and growth of the giant kelp Macrocystis pyrifera. Photosynth. Res..

[47] Bailly J, Coleman JR (1988). Effect of CO(2) concentration on protein biosynthesis and carbonic anhydrase expression in Chlamydomonas reinhardtii. Plant Physiol..

[48] Dionisio-Sese ML, Fukuzawa H, Miyachi S (1990). Light-induced carbonic anhydrase expression in Chlamydomonas reinhardtii. Plant Physiol..

[49] Pollock SV, Colombo SL, Prout DL, Godfrey AC, Moroney JV (2003). Rubisco activase is required for optimal photosynthesis in the green alga Chlamydomonas reinhardtii in a low-CO_2_ atmosphere. Plant Physiol..

[50] Carlberg S, Axelsson L, Larsson C, Ryberg H, Uusitalo J, Baltscheffsky M (1990). Inducible CO_2_ concentrating mechanisms in green seaweeds I. Taxonomical and physiological aspects. Current Research in Photosynthesis.

[51] Wheeler WN (1980). Effect of boundary-layer transport on the fixation of carbon by the giant-kelp Macrocystis pyrifera. Mar. Biol..

[52] Johnston AM, Raven JA (1990). Effects of culture in high CO_2_ on the photosynthetic physiology of Fucus serratus. Br. J. Phycol..

[53] Connell SD, Kroeker KJ, Fabricius KE, Kline DI, Russell BD (2013). The other ocean acidification problem: CO_2_ as a resource among competitors for ecosystem dominance. Philos. Trans. R. Soc. Lond..

[54] Porter ET, Sanford LP, Suttles SE (2000). Gypsum dissolution is not a universal integrator of water motion. Limnol. Oceanogr..

[55] Gerard VA, Mann KH (1979). Growth and production of Laminaria longicruris (Phaeophyta) populations exposed to different intensities of water movement. J. Phycol..

[56] Bivand R, Keitt T, Rowlingson B (2016). Package ‘rgdal’. R Package.

[57] LINZ. LINZ Data Service. https://data.linz.govt.nz/layer/50258-nz-coastlines-topo-150k/history/ Accessed July 2021 (2021).

[58] Kirk JT (1994). Characteristics of the light field in highly turbid waters: A Monte Carlo study. Limnol. Oceanogr..

[59] Strickland JDH, Parsons TR (1968). A Practical Handbook of Seawater Analysis.

[60] Kohler KE, Gill SM (2006). Coral Point Count with Excel extensions (CPCe): A visual basic program for the determination of coral and substrate coverage using random point count methodology. Comput. Geosci..

[61] Axelsson L, Mercado J, Figueroa F (2000). Utilization of HCO3− at high ph by the brown macroalga laminaria saccharina. Eur. J. Phycol..

[62] R Core Team. R: A language and environment for statistical computing. Preprint at (2017).

[63] Bates D, Maechler M, Bolker B, Walker S (2015). Fitting linear mixed-effects models using lme4. J. Stat. Softw..

[64] Hothorn T, Bretz F, Westfall P (2008). Simultaneous inference in general parametric models. Biom. J..

